# The Accuracy of the New Landmark Using Respiratory Jugular Venodilation and Direct Palpation in Right Internal Jugular Vein Access

**DOI:** 10.1371/journal.pone.0103089

**Published:** 2014-07-22

**Authors:** Hyungseok Seo, Dong-Min Jang, Jung-Min Yi, Hong-Gi Min, Jai-Hyun Hwang

**Affiliations:** Department of Anesthesiology and Pain Medicine, Asan Medical Center, University of Ulsan College of Medicine, Seoul, Republic of Korea; University of Palermo, Italy

## Abstract

**Background:**

Although ultrasonography is recommended in internal jugular vein (IJV) catheterization, the landmark-guided technique should still be considered. The central landmark using the two heads of the sternocleidomastoid muscle is widely used, but it is inaccurate for IJV access. As an alternative landmark, we investigated the accuracy of the new landmark determined by inspection of the respiratory jugular venodilation and direct IJV palpation in right IJV access by ultrasonography.

**Methods and Findings:**

Thirty patients were enrolled. After induction of anesthesia, the central landmark was marked at the cricoid cartilage level (M1) and the alternative landmark determined by inspection of the respiratory jugular venodilation and direct palpation of IJV was also marked at the same level (M2). Using ultrasonography, the location of IJV was identified (M3) and the distance between M1 and M3 as well as between M2 and M3 were measured. The median (interquartile range) distance between the M2 and M3 was 3.5 (2.0–6.0) mm, compared to 17.5 (12.8–21.3) mm between M1 and M3. (*P*<0.001) The dispersion of distances between M2 and M3 was significantly smaller than between M1 and M3. (*P*<0.001) The visibility of respiratory jugular venodilation was associated with CVP more than 4 mmHg. Limitations of the present study are that the inter-observer variability was not investigated and that the visibility of the alternative landmark can be limited to right IJV in adults.

**Conclusion:**

The alternative landmark may allow shorter distance for the right side IJV access than the central landmark and can offer advantages in right IJV catheterization when ultrasound device is unavailable.

**Trial Registration:**

Clinical Research Informational Service KCT0000812

## Introduction

Internal jugular vein (IJV) catheterization is commonly performed in clinical practice for central vein access. The central landmark, defined by the apex of the triangle between the two heads of the sternocleidomastoid muscle, is frequently used for IJV catheterization [1]. However, the complications of the central landmark-guided IJV catheterization occur in more than 15% of patients and the central landmark itself was reported to be inaccurate for IJV access [2], [3]. Ultrasonography can reduce the incidence of complications and improves success rate in central venous catheterization (CVC) [4]–[8].

Although ultrasound-guided CVC is recommended as a standard technique, several reasons such as the limited availability of ultrasound machine, lack of training, or longer procedure time can hinder the widespread use of ultrasound device in CVC [6], [9]–[11]. Particularly in emergency situation, which requires urgent CVC without ultrasound device, the landmark-guided technique can be useful and required to be practiced.

Because the central landmark has been known to be inaccurate [3], respiratory jugular venodilation, the pulse-like skin elevation which develops due to IJV dilatation following intrathoracic pressure change in patients receiving positive pressure ventilation, has been suggested as a new landmark especially for right IJV catheterization in patients receiving mechanical ventilation [12]. Additionally, IJV can be palpated directly, which is helpful to identify the IJV location and to increase the accuracy of IJV access.

In the present study, we hypothesized that the alternative landmark determined by the inspection of respiratory jugular venodilation and direct IJV palpation would be closer to the IJV than the central landmark during mechanical ventilation. Although the shorter distance does not mean the better outcomes, better accuracy could reduce the incidence of complications in situation where ultrasonography is limited. For the identification of IJV location, we used ultrasonography and measured the distance between each of the two landmarks and the IJV.

## Subjects and Methods

The protocol for this trial and supporting TREND checklist are available as supporting information; see Checklist S1 and Protocols S1 and S2.

### Subjects

**Figure 1:**
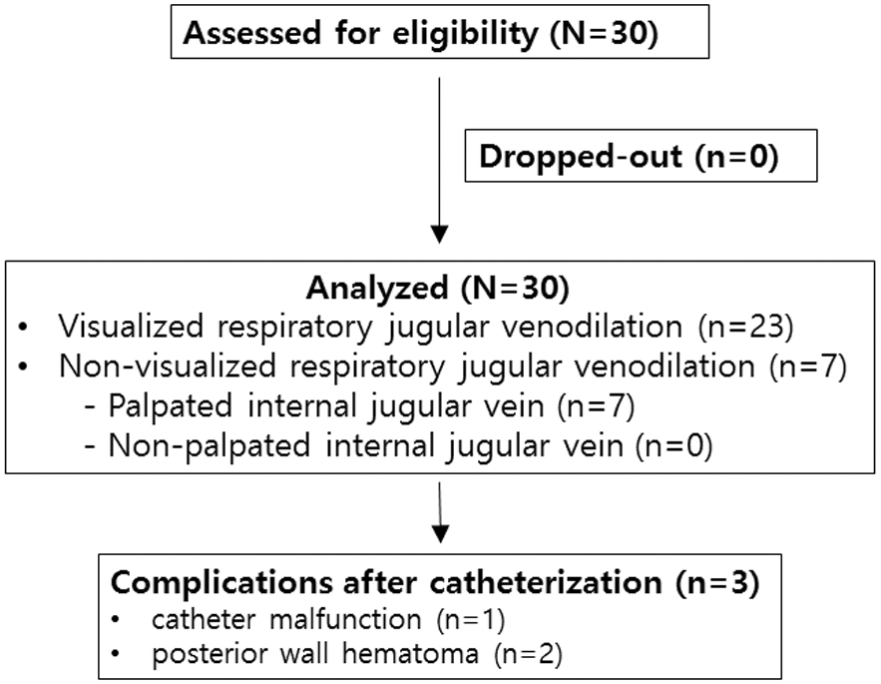
CONSORT flow chart.

Thirty adult patients admitted to the Asan Medical Center for elective urologic surgery between Jan 8 2013 and Jul 1 2013 were enrolled in the study (Figure 1). Patients were excluded from the study if they were aged <20 years, overweight (BMI>30 kg/m^2^), underweight (BMI<18.5 kg/m^2^), with known pulmonary or cardiovascular disorders, or at risk from internal jugular venous catheterization because of limited cervical motion or lesions in head and neck area. In patients who are indicated to receive IJV catheterization and not excluded from the study, randomly sampled patients by operation schedule were participated. Blinding was not accomplished in the present study. The study protocol was approved by the Asan Medical Center Institutional Review Board (approval number: 2012-0859) and registered with the Clinical Research Informational Service, which is the primary registry of WHO International Clinical Trials Registry Platform (KCT 0000812, http://cris.nih.go.kr). Authors obtained the verbal informed consent from all patients participated in the present study. Because authors measured the distance between surface landmark non-invasively and CVC was performed in the standard manner under ultrasonography with no other interventions, ethics committees approved that our study agreed that verbal consent was sufficient. None of the patients were premedicated before the surgery.

### Anesthesia and measurement protocols

Staff anesthesiologists who have fully experienced for both landmark and ultrasound-guided technique were randomly performed IJV catheterization. General anesthesia was induced with thiopental sodium 5 mg/kg, followed by sevoflurane inhalation. Rocuronium bromide 0.6 mg/kg was administered to facilitate tracheal intubation. Anesthesia was maintained with sevoflurane, 50% oxygen and nitrous oxide mixture. Mechanical ventilation was performed with tidal volume of 8 ml/kg, respiratory rate of 10, without positive end-expiratory pressure to maintain end tidal carbon dioxide at 35–40 mmHg. Peak airway pressure was monitored not to exceed 20 cm H_2_O during CVC.

**Figure 2:**
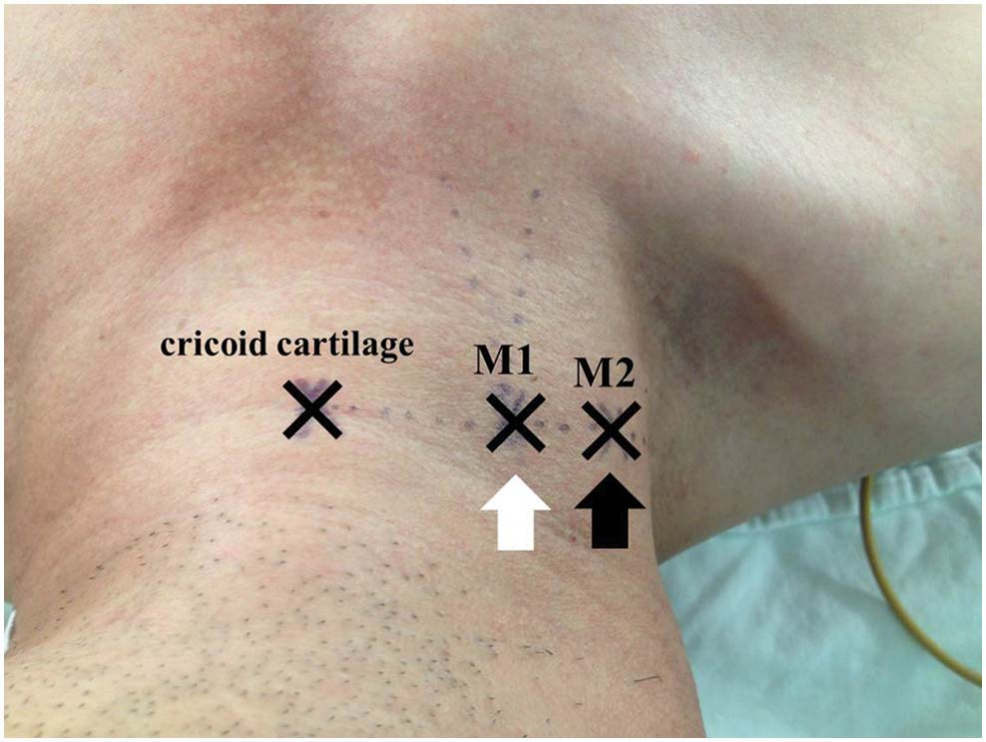
Anatomical location of the two landmarks in the patient. M1 is the central landmark (white arrow), determined by the apex of the triangle between the two heads of the sternocleidomastoid muscle. M2 is the alternative landmark (black arrow), determined by the inspection of respiratory jugular venodilation and direct internal jugular vein palpation.

After induction of anesthesia, patients were placed in a supine, slightly head-down position (approximately 15 degrees) with right arm adduction and the head slightly (approximately less than 15 degrees) turned to the left for the right IJV access. A pillow or facial mask was placed on the left side of the patient’s head to prevent extreme head rotation and overlapping of the IJV and common carotid artery. The central landmark (M1), defined as the apex of the triangle composed of two heads of the sternocleidomastoid muscle and the clavicle, was marked on the line at the level of the cricoid cartilage (Figure 2). At the same cricoid cartilage level containing the central landmark, the alternative landmark (M2) was sought and marked by inspection of the respiratory jugular venodilation, which was identified as a pulse-like skin elevation synchronised with the inspiration phase of positive pressure ventilation [12] and by IJV palpation. After determining the two landmarks, the IJV was identified at the same cricoid cartilage level by using the ultrasound device (S-Nerve, Sonosite Inc., Washington, USA) with a 7.5 MHz linear probe and a cross-sectional view of the IJV was obtained. The ultrasound probe was placed vertical to the skin and adjusted to locate the midpoint of the IJV transverse diameter at the center of the sonographic view. The location of the ultrasound-identified IJV (M3) was marked at the center of the ultrasound probe. As a primary outcome, the distances between M1 and M3, M2 and M3, were measured by a flexible ruler and compared each other. For secondary outcomes, factors associated with the visible respiratory jugular venodilation were investigated.

After sterile skin preparation, the ultrasound-guided CVC was performed by an experienced anesthetist. Before insertion of the puncture needle, the depth and diameter of the IJV, and the diameter of the carotid artery were measured in the same sonographic field of view. After IJV catheterization, the correct catheter location and complications including posterior wall hematoma and pneumothorax was identified by ultrasonography. Central venous pressure (CVP) was also measured after IJV catheterization.

### Sample size calculation and statistical analysis

The pilot data showed that the mean (sd) distances from the central landmark and the alternative landmark to the ultrasound-identified puncture site were 14.3 (3.9) mm and 4.6 (4.2) mm, respectively. We considered the alternative landmark to be within 5.0 mm of the ultrasound-identified puncture site. Assuming a type I error of 0.05 and a desired power of 0.90, 30 patients were required in the present study. Data are presented as mean (sd) in the table and median (interquartile range, IQR) in Figure 2. Statistical analyses were performed using SPSS 13.0 (SPSS Inc. Chicago, IL, USA). A normality test was performed using the Shapiro-Wilk test. Differences in distances were analysed using Mann-Whitney rank sum test. F-test was performed to investigate the difference of the dispersion. Receiver operator characteristic (ROC) curve analysis was performed to determine the predictabilities of CVP, the depth of IJV, and IJV diameter to detect the respiratory jugular venodilation. *P*<0.05 was considered to be statistically significant.

## Results

Thirty patients receiving scheduled urologic surgery were enrolled and none of the patients was excluded. The patient characteristics and anatomic variables are shown in Table 1. Respiratory jugular venodilation was identified in 23 patients (76.7%). In the remaining seven patients, the alternative landmark was identified by direct IJV palpation at the cricoid cartilage level. After the ultrasound-guided IJV catheterization, a case of catheter malfunction and two cases of posterior wall hematoma which are not related with inadvertent arterial puncture developed. There were no complications such as arterial puncture, hemothorax or pneumothorax in all of the patients.

**Table 1:** Patient demographics and sonographic variables.

Patient demographics	Values
Age (years)	60.4 (11.2)
Sex (Male:Female ratio)	20∶10
Body mass index (kg/m^2^)	24.1 (2.6)
**Sonographic variables**	
Depth of internal jugular vein from skin (mm)	8.6 (3.0)
Diameter of internal jugular vein (mm)	11.4 (3.1)
Diameter of common carotid artery (mm)	7.7 (2.0)

Values are expressed as mean (sd) and ratio.

**Figure 3:**
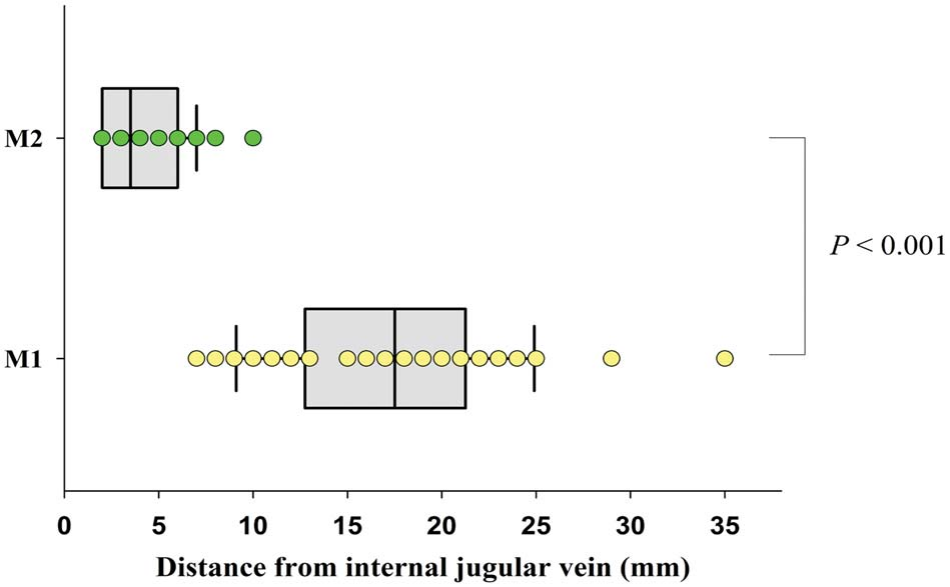
Comparison of distances between M1 and M2 from the ultrasound-identified IJV. Individual and median (interquartile range) distances between M1 and M2 from the ultrasound-identified IJV. For effective representation of the distance, the axes were inverted. The line in the middle box represents the median value and the lateral borders of the box represent interquartile ranges. Individual values are represented as dots (*yellow dots* in M1 and *green dots* in M2). M1, the central landmark; M2, the alternative landmark; IJV, internal jugular vein.

The median [interquartile range] distance between M1 (the central landmark) and M3 (the location of ultrasound-identified IJV) was 17.5 [12.8–21.3] mm and the distance between M2 (the alternative landmark) and M3 was 3.5 [2.0–6.0] mm, (*P*<0.001) (Figure 3). The dispersion of measured distances between M2 and M3 was significantly smaller than between M1 and M3 (F statistic = 8.25, *P*<0.001).

**Figure 4:**
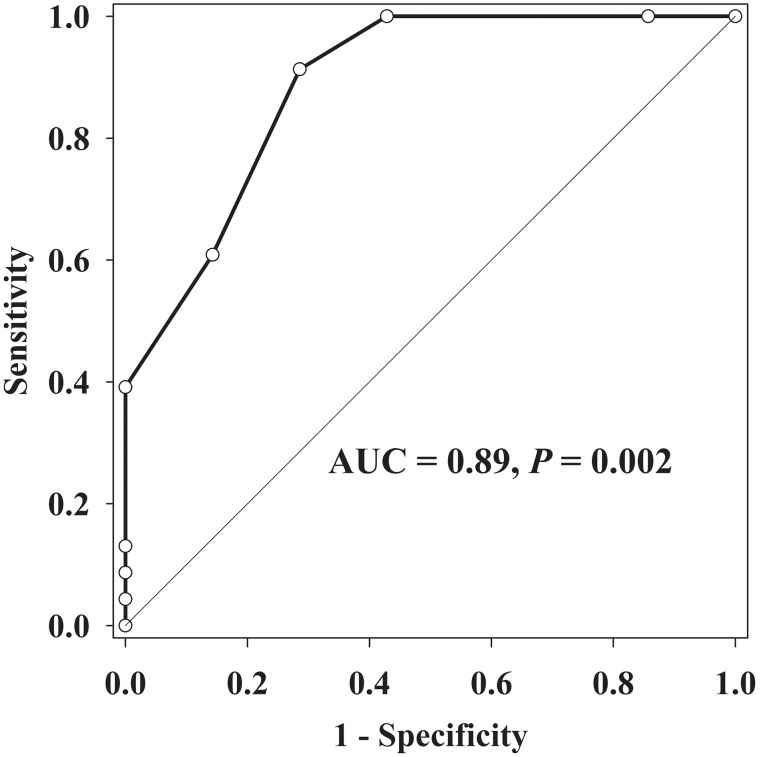
ROC curve of the central venous pressure for the visible respiratory jugular venodilation. Area under the curve is given inside the ROC curve. ROC curve, receiver operator characteristic curve.

ROC curve analysis showed that CVP >4 mmHg was associated with visible respiratory jugular venodilation, demonstrating 91.3% sensitivity and 71.4% specificity. The area under the curve of CVP was 0.89 (*P* = 0.002) (Figure 4). The depth of IJV and IJV diameter was not associated with the visibility of jugular venodilation.

## Discussion

In the present study, we demonstrated that the alternative landmark identified by the inspection of respiratory jugular venodilation and direct IJV palpation was closer to the ultrasound-identified IJV than the central landmark determined by the external anatomy landmark method. Additionally, the visibility of jugular venodilation correlated with CVP >4 mmHg.

A number of techniques for IJV catheterization and the central landmark have been described so far [1], [13]–[15]. The central landmark is the apex of the triangle defined by the two heads of the sternocleidomastoid muscle and the clavicle, when the patient’s head is turned away from the catheterization site. However, it has been associated with more frequent complications than ultrasound-guided CVC [6], [16], [17], and the inaccuracy of the central landmark might contribute to the risk of arterial puncture and failure to find IJV [3]. The ultrasound-guided CVC is beneficial in improving success rate and reducing the incidence of complications such as arterial puncture, pneumothorax, and catheter-associated blood stream infection [16], [17]. Therefore, the use of real-time ultrasound-guided CVC has been commonly recommend in clinical practice [4], [5], [7], [8], [11].

Although ultrasound-guided CVC has been recommended as a standard practice, some studies have shown that the use of ultrasound during CVC still remains limited in clinical practice. The limited availability and the physician’s perceived utility of ultrasound devices contribute to their limited use [9], [10], [18]. In addition to these limiting factors, there is a potential risk of operators losing the skills necessary for the landmark technique. In emergency situations when CVC should be performed without time delay, the landmark technique might be beneficial because it requires less time for preparation and vein puncture. Moreover, the complication rate was reported to be similar between landmark technique and ultrasound-guided technique [19], [20]. For these reasons, the landmark technique in CVC should still be considered as a clinically acceptable practice, despite the recommendation of ultrasound-guided CVC as a standard practice. Additionally, Health Technology Assessment also recommends that training for CVC using the landmark technique should be given in addition to the training for ultrasound-guided CVC [6].

However, ultrasonography revealed that the central landmark was unreliable for accessing the IJV and head rotation during the central landmark guided CVC increased the overlap of the carotid artery and IJV, leading to increased inadvertent arterial puncture [21], [22]. These findings suggest that the alternative landmark can be considered to access IJV in landmark-guided CVC, especially when ultrasound is not available.

Before the inspection of the respiratory jugular venodilation, we standardized the CVC protocol. Patients were maintained with supine, slightly head-down position to increase IJV diameter [23], [24]. Respiratory jugular venodilation, which is the increase in IJV diameter caused by intrathoracic pressure change synchronized with respiration, can be easily observed in patients receiving positive pressure ventilation, and it reflects the location of IJV more accurately than the central landmark, particularly in right side IJV catheterization [12], [25]. When jugular venodilation was visible, the success rate of IJV catheterization at first attempt and arterial puncture incidence was 79% and 0.6% respectively, compared to 42.9% and 9.5% when jugular venodilation was not visible [12]. Since respiratory jugular venodilation represents the change in IJV diameter, it can indicate the location of the right IJV directly. Additionally, direct palpation may help to identify the location of the right IJV and it may increase the accuracy of IJV identification in combination with the inspection of jugular venodilation. We observed jugular venodilation in 77% of the patients and palpated the IJV directly in patients lacking the visible jugular venodilation. The median distance of the alternative landmark from the IJV was 3.5 mm, suggesting that the position of the alternative landmark is nearly identical to the position of IJV because the mean IJV diameter was 17.5 mm. In addition to the small median value, the small range of the distance from ultrasound-identified IJV also supports the notion that the alternative landmark is more accurate for accessing the IJV than the central landmark in CVC.

The respiratory jugular venodilation was visible in more than 75% of ventilated patients, however, its visibility was not associated with the level of the operator’s experience or the IJV size, but with the extent of the dynamic change of IJV during the respiratory cycle [26]. In the present study, the ROC curve analysis showed the visibility of jugular venodilation was associated with the CVP and the CVP of more than 4 mmHg could predict the visible jugular venodilation (area under curve = 0.89, *P* = 0.002). This finding suggests that low CVP status would be less influenced by intrathoracic pressure change during mechanical ventilation, leading to small dynamic change of IJV.

The alternative landmark-guided IJV catheterization has several limitations. First, the variability of the alternative landmark between different investigators was not compared. Since there is no exact definition of the findings of the jugular venodilation and IJV palpation, the location of the alternative landmark might be influenced by investigator’s experience. However, it was technically difficult to keep blinding between investigators in the present study design and the use of an ultraviolet ray-sensitive pen might be helpful for the blinding. Second, the respiratory jugular venodilation cannot be observed in all cases. In normal spontaneous breathing, respiratory movement with negative interpleural pressure can collapse internal jugular vein, thus respiratory jugular venodilation can be observed in patients receiving positive pressure ventilation [27]. Although it is commonly observed in approximately 80% of ventilated patients, there is a lack of evidence for its efficacy in paediatric patients. Moreover, respiratory jugular venodilation is not clearly identified in left IJV, because the left IJV is relatively far from the right atrium. Therefore, the efficacy of the alternative landmark can be limited to right IJV access in adult patients receiving positive pressure ventilation. Third, we did not investigate the effectiveness of the alternative landmark, such as the success rate at first attempt and the incidence of arterial puncture, in IJV catheterization. Although the distance of the alternative landmark from the IJV is shorter than that of the central landmark, the shorter distance might not mean better. To compare the efficacy between the two landmarks in IJV catheterization, a further study should be required. However, there can be an ethical problem of not using ultrasonography in CVC because the ultrasound-guided technique is currently recommended as a standard procedure. Although we performed IJV catheterization under ultrasonography guidance, previous research of IJV catheterization using jugular venodilation with 30 degrees left-turned head position and needle directed caudad with 60 degrees of puncture angle, revealed increase of the success rate at first attempt and decrease the arterial puncture incidence [12].

In summary, the alternative landmark identified by respiratory jugular venodilation and IJV palpation may allow more accuracy for the right side IJV access than the central landmark identified by the sternocleidomastoid muscle. To reduce the complications related to CVC, ultrasound-guided technique is inevitable. But, for access to right internal jugular vein when an ultrasound device is not available, better accuracy can reduce complication rate and the alternative landmark-guided technique may offer advantages.

## Supporting Information

Checklist S1 **Trend Statement Checklist.**
(PDF)

Data S1 **Clinical Research Data.**
(XLSX)

Protocol S1 **Clinical Research Protocol (English).**
(DOCX)

Protocol S2 **Clinical Research Protocol (Korean).**
(PDF)
