# Supplementary material for: The Accuracy of the New Landmark Using Respiratory Jugular Venodilation and Direct Palpation in Right Internal Jugular Vein Access
Source: PLoS One. 2014 Jul 22;9(7):e103089. doi: 10.1371/journal.pone.0103089 (PMC4106888; doi:10.1371/journal.pone.0103089)
Supplement: Protocol S1 — Clinical Research Protocol (English). (DOCX) [file pone.0103089.s003.docx]

**Clinical Research Protocol**

**Title**

**An alternative landmark using combined jugular venodilation and palpation is more accurate to the insertion point under ultrasound guidance than the central landmark in internal jugular venous catheterization**

**Corresponding author**

Min Hong-Gi, Clinical assistant professor

**Co-authors**

Seo Hyungseok, Clinical assistant professor

Kim Ji-Yeon, Resident

Moon Young-Jin, Resident

Submission date: 2012 -11- 20

**.**

| **Title** | An alternative landmark using combined jugular venodilation and palpation is more accurate to the insertion point under ultrasound guidance than the central landmark in internal jugular venous catheterization |
| --- | --- |
| **Purpose** | During internal jugular venous catheterization, ultrasound-guided technique can be recommended as the standard because of it high success rate and decreased the risk of posterior wall puncture. However, limited availability of ultrasound machine or lack of skill can hinder the widespread of the ultrasound use. Therefore, the landmark-guided catheterization cannot be excluded and there is still a need to increase the accessibility in internal jugular vein catheterization. The central landmark using landmark-guided technique has been known to be inaccurate to the true location of internal jugular vein, thereby being related catheterization related complications. In the present study, we suggested an alternative landmark using combination of the inspection of respiratory jugular venodilation and direct internal jugular vein palpation. And we investigated the distance between the alternative landmark and ultrasound identified internal jugular vein and compared with that of the central landmark. |
| **Principal Investigator** | Min Hong-Gi |
| **Institute** | Asan Medical Center |
| **Sponsor** | None |
| **Cohort group** | 1. Patients who are indicated to internal jugular venous catheterization during general abdominal or neurological surgery 2. Adults aged 21 or more 3. Patients of class 1 or 2 in American Society of Anesthesiology classification. 4. Patients without head and neck disease, or without clinically important cardiopulmonary problems |
| **Clinical trial of drug** | none |
| **Study design** | An prospective observation study. After induction of anesthesia, we measure the distance between ultrasound-identified internal jugular vein and two different landmarks. |

2. Summary

| **Measurement** | After general anesthesia induction, patient is placed in a supine position with slightly rotated to left. The central landmark (M1), defined as the apex of the triangle composed of two heads of the sternocleidomastoid muscle and the clavicle, was marked on the line at the level of the cricoid cartilage. And the alternative landmark (M2) using the inspection of respiratory jugular venodilation and the direct internal jugular vein palpation was marked at the same level. The true location of internal jugular vein (M3) was identified with ultrasonography. Finally, the distance between M1 and M3 was compared with the distance between M2 and M3. |
| --- | --- |
| **Statistical analysis** | - Data are presented as mean±sd or median (interquartile range). - Demographics or measured data were analysed using Mann-Whitney rank sum test or *t*-test. - *P* < 0.05 was considered to be statistically significant. |

**3. Table of Contents**

1. Cover page ................................................................................................................................ 1

2. Summary.................................................................................................................................... 2

3. Table of Contents....................................................................................................................... 4

4. Ethics……................................................................................................................................. 5

5. Backgrounds ............................................................................................................................. 6

6. Expected periods of research......................................................................................................7

7. Subjects and Methods................................................................................................................ 8

8. References.................................................................................................................................12

**4. Ethics**

The present study is conducted by the research protocol approved by Asan Medical Center Institutional Review Board and according to the principles expressed in the Declaration of Helsinki. Authors must protect the private information of subjects participating in the present study.

**5. Backgrounds**

During internal jugular venous catheterization, ultrasound-guided technique can be recommended as the standard because of it high success rate and decreased the risk of posterior wall puncture. However, limited availability of ultrasound machine or lack of skill can hinder the widespread of the ultrasound use. Therefore, the landmark-guided catheterization cannot be excluded and there is still a need to increase the accessibility in internal jugular vein catheterization. The landmark-guided technique has been known to be inaccurate to the true location of internal jugular vein, thereby being related catheterization related complications.

For landmark-guided internal jugular venous catheterization, the central landmark defined by the two heads of sternocleidomastoid muscle has been used traditionally. However, the central landmark does not correlate with internal jugular vein and it results in complications related with catheterization. Therefore, we suggested an alternative landmark using combination of the inspection of respiratory jugular venodilation and direct internal jugular vein palpation in the present study. Respiratory jugular venodilation can be seen easily and it suggests the direct location of internal jugular vein. Moreover, internal jugular vein can be directly palpated, which can help to identify internal jugular vein location in the surface anatomy. We investigated the distance between the alternative landmark and ultrasound identified internal jugular vein and compared with that of the central landmark.

**6. Expected periods of research**

From the approval of Institutional Review Board to 25, Nov, 2013

**7. Subjects and Methods**

**7.1 Subjects**

**7.1.1 Inclusion criteria**

1) Patients who are indicated to internal jugular venous catheterization during surgery

2) Adults aged 21 or more

3) Patients of class 1 or 2 in American Society of Anesthesiology classification

4) Patients without head and neck disease, or without clinically important cardiopulmonary problems

**7.1.2 Exclusion criteria**

1) Abnormal findings on preoperative pulmonary function test

2) Body mass index > 30 or < 18.5

3) Patients with severe decreased cardiopulmonary function

4) Patients with head and neck disease, who are difficult to internal jugular venous catheterization

5) Patients who cannot use internal jugular vein because of neck surgery or other medical conditions

**7.2. Statistical analysis**

**7.2.1 Statistics**

1. Data are presented as mean±sd or median (interquartile range)
2. Demographics or measured data were analysed using Mann-Whitney rank sum test or *t*-test.
3. *P* < 0.05 was considered to be statistically significant.

**7.2.2 Sample size**

Thirty patients who are suitable for the inclusion criteria and are not suitable for the exclusion criteria

**7.2.3 Sample size**

In a pilot study, the distance between the conventional landmark and ultrasound-identified internal jugular vein was 1.4±0.39 cm, and between the alternative landmark and internal jugular vein was 0.46±0.41 cm. Using this result, we hypothesized that the distance between the alternative landmark and the internal jugular vein was less than 0.4 cm. In the set of type I error of 0.05 and desired power of 0.9, we calculated 24 subjects for total sample size. In consideration of data loss or measurement error, we enrolled 30 subjects for the present study.

**7.3 Methods**

**7.3.1 Study design**

A prospective observation study performed in single institute.

**7.3.2 Study methods**

After induction of anesthesia, patients were placed in a supine position with right arm adduction and the head slightly turned to the left for the right internal jugular vein (IJV) access. A pillow or facial mask was placed on the left side of the patient’s head to prevent extreme head rotation and overlapping of the IJV and common carotid artery. The central landmark (M1), defined as the apex of the triangle composed of two heads of the sternocleidomastoid muscle and the clavicle, was marked on the line at the level of the cricoid cartilage. At the same cricoid cartilage level containing the central landmark, the alternative landmark (M2) was sought and marked by inspection of the respiratory jugular venodilation, which was identified as a pulse-like skin elevation synchronised with the inspiration phase of positive pressure ventilation and by IJV palpation. After determining the two landmarks, the IJV was identified at the same cricoid cartilage level by using the ultrasound device and a cross-sectional view of the IJV was obtained. The ultrasound probe was placed vertical to the skin and adjusted to locate the midpoint of the IJV transverse diameter at the center of the sonographic view. The location of the ultrasound-identified IJV (M3) was marked at the center of the ultrasound probe. The distances between M1 and M3, M2 and M3, were measured by a flexible ruler.

M2, alternative landmark

M1, central landmark


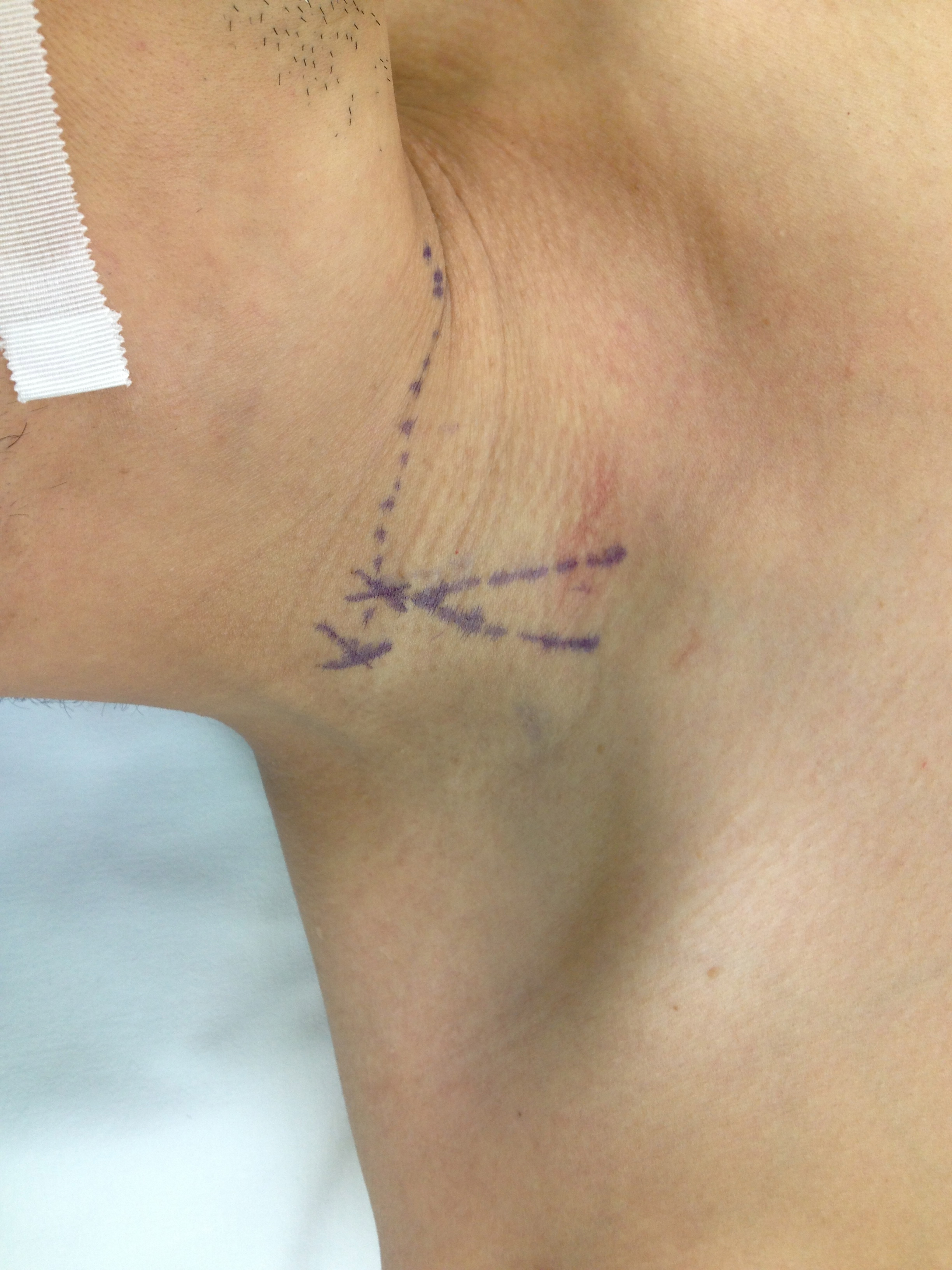


**7.3.3 Randomization and Blindness**

Subjects are sampled randomly by operation schedule. The present study does not meet blindness.

**7.3.4 Patients Safety**

Because internal jugular venous catheterization in the present study is performed with ultrasonography and authors measure the distance on surface anatomy, the risk of the patient was not increased compared with the standard central venous catheterization technique. The anticipated risks of internal jugular venous catheterization include carotid artery puncture, hematoma, or pneumothorax. By using ultrasonography, we can find the catheterization related complications immediately after procedure.

**7.3.5 Data analysis**

Authors record the clinical data to our Clinical Research Form and plan to edit data by Microsoft Excel. All data are analyzed with Sigmaplot 12.0

**7.3.6 The safety of patients**

The present study has no further or increased risk of complications compared with ultrasound-guided internal jugular catheterization. Moreover, authors have great concerns to protect private information of the patient during the entire research period.

**7.3.7 Expected complications**

The patients receiving internal jugular venous catheterization can have general risks including puncture site pain, catheter-related infection, localized edema, common carotid artery puncture, and hemo/pneumothorax.

To protect from catheter-related infection, catheterization should be performed with sterile and standardized manner. When carotid artery is punctured, needle should be removed immediately and puncture site should be compressed for more than 5 minutes. When hemo/pneumothorax is clinically suspected after catheterization, chest radiography is obtained and proper management is given immediately.

**7.3.8 Cessation or drop out of study**

The present study is a non-invasive observational study and does not require the confirmative drop-out criteria. However, when ultrasonography reveals that the internal jugular vein diameter is smaller than the common carotid artery diameter, non-palpated non-visualized jugular vein, or abnormal anatomical course of internal jugular vein, the patient can be dropped out of the study.

**8. Reference**

1. Peter L. Bailey, Laurent G. Glance, Michael P. Eaton, Bob Parshall, Scott McIntosh A Survey of the Use of Ultrasound During Central Venous Catheterization. Anesth Analg 2007;104:491-7
2. A.C.Gordon, J.C.Saliken, D.Johns, R.Owen, R.R.Gray US-guided Puncture of the Internal Jugular Vein: Complications and Anatomic Considerations
3. T.Lim, H-G.Ryu, C-W.Jung, Y. Jeon, J-H.Bahk Effect of the bevel direction of puncture needle on success rate and complications during internal jugular vein catheterization. Crit Care Med 2012; 40:491-494
4. H. Hayashi, C. Ootaki, M. Tsuzuku and M. Amano Respiratory jugular venodilation: A new landmark for right internal jugular vein puncture in ventilated patients. Journal of Cardiothoracic and vascular anesthesia 2000; 14; 40-44
