# Supplementary material for: The Accuracy of the New Landmark Using Respiratory Jugular Venodilation and Direct Palpation in Right Internal Jugular Vein Access
Source: PLoS One. 2014 Jul 22;9(7):e103089. doi: 10.1371/journal.pone.0103089 (PMC4106888; doi:10.1371/journal.pone.0103089)
Supplement: Protocol S2 — Clinical Research Protocol (Korean). (PDF) [file pone.0103089.s004.pdf]

임상연구계획서

<u>연구 제목</u>

내경정맥을 통한 중심정맥관 삽입술에서 초음파를 이용하여 기존의 표지자에 의한 자입점과 경정맥 확장 및 촉지에 의한 자입점의 정확도 비교

An alternative landmark using combined jugular venodilation and palpation is more accurate to the insertion point under ultrasound guidance than the central landmark in internal jugular venous catheterization

<u>책임 연구자</u>

서울아산병원 마취통증의학과 임상조교수 민홍기

<u>공동 연구자</u>

서울아산병원 마취통증의학과 임상강사 서형석

서울아산병원 마취통증의학과 전공의 김지연

서울아산병원 마취통증의학과 전공의 문영진

연구 계획서 제출일: 2012년 11월 20일

.

2. 연구계획서요약

**제 목**	내경정맥을 통한 중심정맥관 삽입술에서 초음파를 이용하여 기존의 표지자에 의한 자입점과 경정맥 확장 및 촉지에 의한 자입점의 정확도 비교
**목 적**	내경정맥을 통한 중심정맥관 삽입시 초음파유도하에서 내경정맥으로 접근하는 방법은 기존의 맹목하 접근법에 비해 성공률이 높고, 후벽천자와 관련된 합병증이 감소하기 때문에 표준적인 방법으로 이용된다. 하지만 중심정맥관 삽입술 시 임상적인 상황에 따라 초음파장비를 이용하기 어려울 수 있을 뿐 아니라 초음파감시에 따른 비용이 증가할 수 있기 때문에 기존의 표지자에 의한 맹목하 삽입술을 배제할 수는 없다. 하지만 맹목하 삽입술에서 결정되는 자입점과 실제 내경정맥의 위치가 일치하는 것은 아니기 때문에 맹목하 삽입술에서도 보다 정확한 자입점을 찾는 것이 필요하다. 본 연구에서는 중심정맥관 삽입술 시에 기존에 사용되던 표지자에 대한 자입점과, 경정맥 확장을 관찰하고 실제로 경정맥을 촉지하여 결정한 자입점의 위치관계를 초음파를 통하여 비교하고 어떤 방법이 더 실제 내경정맥의 위치와 유사한지 확인하고자 한다.
**임상시험 책임자**	민 홍 기
**연구기관**	서울아산병원 마취통증의학과
**의뢰자/모니터**	없음
**대상 피험자**	1) 서울아산병원에서 예정된 일반외과 수술 중 내경정맥을 통한 중심정맥관 삽입에 적응이 되는 환자<br>2) 만 21세 이상의 성인<br>3) 미국마취과학회 신체분류상 1, 2등급환자 (ASA PS 1 또는 2)<br>4) 경추 및 두경부 질환이 없고 심폐기능의 손상이 없는 환자.
**임상시험약**	없음
**시험 설계**	서울아산병원 수술장에서 시행되는 정규수술 중, 일반외과 수술에서 중심정맥관 삽입에 적응증이 되는 환자를 대상으로 시행한다. 중심정맥관 삽입술을 시행 전 표지자에 의한 자입점과 경정맥의 관찰과 촉진을 통해 결정한 자입점의 위치를 표시하고 초음파를 이용해 실제 경정맥의 위치와의 거리를 측정한다.

**평가방법**	환자의 유상연골 높이 또는 표지자에 의한 자입점과 같은 높이에서 호흡에 따른 경정맥확장을 관찰하고 경정맥을 촉진하여 새로운 자입점을 표시한다. 이후 초음파를 사용하여 확인한 내경정맥의 위치와 두 자입점간의 수평거리를 측정한다.
**통계분석**	• 모든 자료는 평균 ± 표준편차와 빈도 수 (%)로 표현한다.<br>• 역학자료 및 수집된 자료는 unpaired t-test를 이용하여 비교한다.<br>• P값이 0.05미만이면 통계적으로 유의한 것으로 한다.

3.목차

1.표지.. 1

2.연구계획서요약.. 2

3.목차.. 4

4.윤리적 고려.. 5

5.배경 및 목적.. 6

6.예상연구기간... 7

7.대상 및 방법.. 8

8.참고 문헌...12

4. 윤리적 고려

본 시험은 서울아산병원 임상연구심의위원회(IRB)의 승인을 받은 시험계획서에 따라 진행되고 헬싱키선언에 근거하여 KGCP 및 ICH 가 정한 임상시험관리기준(GCP)에 준하여 실시될 것이며, 대상환자의 비밀을 철저히 유지할 것이다.

5. 배경 및 목적

내경정맥을 통한 중심정맥관 삽입시 초음파유도하에서 내경정맥으로 접근하는 방법은 기존의 맹목하 접근법에 비해 성공률이 높고, 후벽천자와 관련된 합병증이 감소하기 때문에 표준적인 방법으로 이용된다. 하지만 중심정맥관 삽입술 시 임상적인 상황에 따라 초음파장비를 이용하기 어려울 수 있을 뿐 아니라 초음파 감시에 따른 비용이 증가할 수 있다. 또한 개인마다 초음파를 사용하는 기술적 수준에 차이를 보일 수 있고, 초음파를 이용하더라도 합병증을 배제할 수는 없다. 따라서 중심정맥관의 삽입에서 기존의 표지자에 의한 맹목하 삽입술을 배제할 수는 없다. 하지만 맹목하 삽입술에서 기존의 표지자에 의해 결정되는 자입점(흉쇄유돌근의 양측 두부가 갈라지는 점)과 실제 내경정맥의 위치가 일치하는 것은 아니기 때문에 맹목하 삽입술을 시행하더라도 보다 정확한 자입점을 찾는 것이 중심정맥관 삽입에 대한 합병증을 줄이기 위해 필요하다.

내경정맥을 확인하기 위해 직접 관찰하는 방법과 실제로 만져보는 방법이 이용되는데, 호흡 등에 따라 경정맥이 확장되는 것을 관찰할 수 있고 실제로 경정맥을 촉지할 수도 있다. 실제 경정맥의 위치를 파악하여 천자한다면 같은 맹목하 삽입술이라고 하더라도 합병증의 빈도가 감소할 수 있고 혈관 손상 등의 가능성이 적을 수 있다. 본 연구에서는 중심정맥관 삽입술 시에 기존에 사용되던 표지자에 대한 자입점과, 경정맥 확장을 관찰하고 실제로 경정맥을 촉지하여 결정한 자입점의 위치관계를 초음파를 통하여 비교하고 어떤 방법이 더 실제 내경정맥의 위치와 유사한지 확인하고자 한다

6. 예상 연구 기간

IRB 승인일로부터 2013년 11월 25일까지

7. 대상 및 방법

7.1 연구대상

피험자 선정기준에 합당하고 제외기준에 부합하지 않는 환자

7.1.1 선정기준

다음 기술된 조건에 부합되는 환자들을 선정한다.

1) 서울아산병원에서 예정된 일반외과 및 신경외과 수술 중 내경정맥을 통한 중심정맥관 삽입에 적응이 되는 환자
2) 만 21세 이상의 성인남녀
3) 미국마취과학회 신체분류상 1, 2등급환자 (ASA PS 1 또는 2)
4) 경추 및 두경부 질환이 없고 심폐기능의 손상이 없는 환자.

7.1.2 제외기준

다음의 기술된 조건에 해당되는 환자는 제외한다.

1) 수술전 폐기능 검사에서 심각한 이상소견을 보이는 환자
2) 체질량지수 30 초과의 고도비만 환자 또는 18.5 미만의 저체중 환자
3) 기타 심각한 심폐질환, 뇌혈관질환을 동반하는 환자
4) 경추질환 또는 두경부 질환으로 내경정맥 천자가 어려운 경우
5) 수술 또는 동반질환 등의 이유로 우측 내경정맥을 사용할 수 없는 환자

7.2. 통계분석 방법과 표본산출 근거

7.2.1 통계분석 방법

- 모든 자료는 평균 ± 표준편차와 빈도(%)로 표현한다.
- 환자의 역학자료, 술 중 자료는 unpaired t-test를 이용하여 비교한다.
- P값이 0.05미만이면 통계적으로 유의한 것으로 한다.

7.2.2 피험자수

대상기준을 만족하고 제외기준을 만족하지 않는 피험자 30명을 대상으로 한다.

7.2.3 설정근거

예비실험에서 초음파 유도하 conventional landmark와 alternative landmark가 ultrasound guided puncture site와 각각 1.4 (0.39), 0.46 (0.41)의 거리를 보였다. 이를 근거로 alternative landmark가 puncture site와 0.4 cm 미만의 차이를 보이는 것을 가정하여. α 값을 0.05, desired power를 0.9로 설정하여 계산된 표본 수는 24 명이었다. 자료수집 및 측정 과정에서 발생할 수 있는 손실을 고려하여 30명을 피험자로 선정한다.

7.3 임상시험의 방법

7.3.1 임상시험의 설계

본 임상시험은 단순관찰연구로, 선정기준에 해당하고 제외기준에 해당하지 않는 환자를 대상으로 중심정맥관을 삽입하는 과정에서 시행한다.

7.3.2 실험방법

중심정맥관을 삽입하는 방법은 초음파 유도하에서 시행한다. 마취 유도 후 앙와위에서 환자의 경부를 좌측으로 10~15도 정도 돌리고 양 팔을 몸에 붙인 후 시행한다. 통상적인 중심정맥관 삽입과 동일한 방법으로 멸균한 후 멸균된 skin marker를 이용하여 기존의 흉쇄유돌근의 두부가 갈라지는 지점 또는 윤상연골의 높이를 기준으로 하여 자입점을 표시한다. (자입점1, conventional landmark) 자입점1과 동일한 높이에서 환자의 경부를 자세히 관찰하여 호흡에 따른 경정맥확장이 관찰되고 경정맥이 촉진되는 곳을 찾아 자입점을 표시한다. (자입점2, alternative landmark) 초음파를 이용하여 탐침의 한가운데 경정맥의 중앙이 오도록 한 후 해당 위치를 표시한다. (기준점)

멸균된 줄자를 이용하여 기준점과 자입점1, 기준점과 자입점2, 자입점1과 자입점2의 거리를 기록한다. 이후로는 통상적인 초음파 유도하에 중심정맥관 삽입하는 방법을 따르도록 한다.

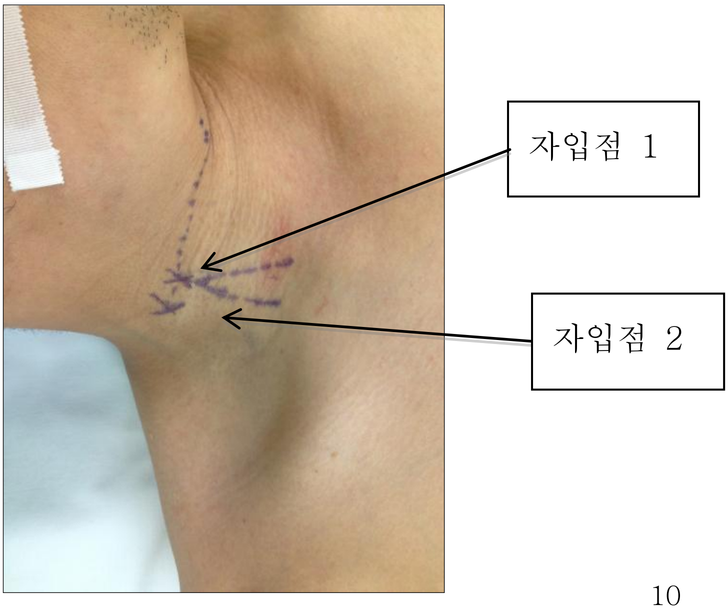

7.3.3 무작위배정과 눈가림법

본 연구는 중심정맥관 삽입의 적응증이 되는 환자를 대상으로 하는 단순관찰연구로 무작위배정이 해당되지 않고 연구설계상 눈가림법도 해당되지 않는다.

7.3.4 안정성 평가

본 연구는 초음파를 통한 해부학적 위치관계를 파악하는 것으로 실제 중심정맥관 삽입술은 초음파를 사용하여 시행하기 때문에 통상적인 중심정맥관 삽입술의 안전성과 유사하다. 예상할 수 있는 위험으로는 내경동맥천자, 기흉, 혈종 등이 있다. 초음파를 사용하는 경우 상기 합병증들을 즉각 확인할 수 있으며, 맹목하에서 시행하는 경우라도 시진, 청진 등을 통해서 합병증을 확인할 수 있다.

7.3.5 데이터베이스 구축 및 자료분석계획

증례기록지에 기록한 임상자료를 MS Excel 프로그램을 이용하여 정리하고, SPSS 또는 SigmaPlot 등의 통계프로그램을 이용하여 분석할 계획이다.

7.3.6 피험자 안전보호에 관한 대책

본 연구는 단순 관찰연구로 기존의 초음파 유도하에 삽입하는 방법에 대한 변형이 없기 때문에 피험자의 위험은 통상적인 중심정맥관 삽입의 위험과 다르지 않다. 기존의 연구에서 초음파를 이용한 중심정맥관 삽입이 기존의 맹목천자법에 비해 합병증이 훨씬 적은 것으로 알려져있다. 또한, 연구자는 연구과정에서 피험자의 개인정보, 사생활과 관련된 정보가 노출되지 않도록 노력할 것이다.

7.3.7 피험자에게 예측되는 부작용

피험자에게는 중심정맥관 삽입에 따른 합병증인 삽입 부위의 통증 및 감염, 국소부종, 경동맥천자가능성, 기흉 및 혈흉 등이 발생할 수 있으나 이는 중심정맥관 삽입에 따른 일

반적인 합병증으로, 본 연구에서 피험자에게 예측되는 부작용은 일반적인 합병증의 위험도와 다르지 않다.

시술부위의 감염을 예방하기 위하여 베타딘 등으로 천자부위를 소독하고 멸균된 세트를 사용한다. 내경동맥을 천자하는 경우는 즉시 천자바늘을 제거하고 해당 부위를 5~10분간 압박하여 지혈한다. 시술 후 기흉이나 혈흉 등이 의심되는 경우 흉부촬영을 시행하고 범위에 따라 흉관삽입 등 적절한 조치를 취하도록 한다.

7.3.8 실험의 중지 및 탈락기준

본 연구는 비침습적인 관찰연구로 연구과정에서 중지기준은 필요없을 것으로 생각된다.

초음파로 관찰하여 피험자의 내경정맥 직경이 경동맥 직경보다 작은 경우, 촉진이 어렵거나 명확한 경정맥 확장이 관찰되지 않는 경우라면 수집대상에서 제외한다. 또한 초음파 검사상 내경정맥의 주행이 일반적이지 않는 경우도 자료수집대상에서 제외한다.

8. 참고문헌

1. Peter L. Bailey, Laurent G. Glance, Michael P. Eaton, Bob Parshall, Scott McIntosh A Survey of the Use of Ultrasound During Central Venous Catheterization. Anesth Analg 2007;104:491-7
2. A.C.Gordon, J.C.Saliken, D.Johns, R.Owen, R.R.Gray US-guided Puncture of the Internal Jugular Vein: Complications and Anatomic Considerations
3. T.Lim, H-G.Ryu, C-W.Jung, Y. Jeon, J-H.Bahk Effect of the bevel direction of puncture needle on success rate and complications during internal jugular vein catheterization. Crit Care Med 2012; 40:491-494
4. H. Hayashi, C. Ootaki, M. Tsuzuku and M. Amano Respiratory jugular venodilation: A new landmark for right internal jugular vein puncture in ventilated patients. Journal of Cardiothoracic and vascular anesthesia 2000; 14; 40-44
